# Reconsidering the value of Caprini scores and venous thromboembolism (VTE) risk mitigation methods in plastic surgery patients

**DOI:** 10.1007/s00266-023-03261-x

**Published:** 2023-01-20

**Authors:** Eric Swanson

**Affiliations:** Swanson Center, 11413 Ash St, Leawood, KS 66211 USA

*Level of Evidence V* This journal requires that authors assign a level of evidence to each article. For a full description of these Evidence-Based Medicine ratings, please refer to the Table of Contents or the online Instructions to Authors www.springer.com/00266

In their recent simulation study, Pannucci et al. [1] report Caprini scores among 100 patients consulting for breast augmentation. Of course, the venous thromboembolism (VTE) rate would be expected to be zero in a group size this small [1, 2]. For the purpose of the simulation, the relative VTE risk for patients with Caprini scores ≥ 7 (a 5.96-fold increase) was extrapolated from the VTE Prevention Study data for plastic surgery inpatients [3], which would be unlikely to include many breast augmentations. The authors recognize that inpatient data are not directly relatable to the ambulatory population [1]. Regardless, if the risk of VTE in a breast augmentation patient is 0.01% or 0.02% [1], a 5.96-fold increase in risk represents a VTE risk of about 1:1,000 in patients deemed to be at higher risk. Only four patients had Caprini scores of 7 or 8. The authors’ simulation shows that most VTEs (80%) are likely to occur in patients with Caprini scores ≤ 6 [1].

Despite its negligible predictive value, the authors consider risk stratification a rapid and low-cost strategy that may help to prevent a very rare catastrophic event [1]. They compare this method to wearing seat belts while driving, or finding the needle in the haystack [1]. The authors believe that patients deemed to be at higher risk may benefit from risk modification or additional medical workup [1]. Unlike in previous publications [2, 3], chemoprophylaxis is not mentioned. The authors reference a favorable risk/benefit ratio for anticoagulation in surgery inpatients with Caprini scores ≥ 7, but decline to offer chemoprophylaxis to aesthetic surgery outpatients [1], recognizing the difference in patient characteristics.

It is reasonable to ask, if Caprini scores are not used in deciding whether to prescribe chemoprophylaxis, what is the point of calculating one? Pannucci [2] believes that these scores serve as a “jumping-off point for surgeons to consider and conceptualize VTE risk among the aesthetic population.” The authors identified 28 women (28%) who they believe had at least one potentially modifiable risk factor or risk factor potentially benefiting from further investigation before surgery [1], selecting hormone supplementation as the most common modifiable risk factor. Oral contraceptives were used by 18 women (18%). Five patients had a family history of VTE, which is not a modifiable risk factor [2]. The five remaining patients had a history of lost pregnancies, genetic hypercoagulability, varicose veins, or a personal history of VTE. The authors do not report whether their breast augmentation patients were instructed to stop taking their birth control pills or whether any were referred for coagulation studies or a hematology consultation [2]. None of the patients was denied surgery based on perceived VTE risk [1].

When discussing the possible benefit of risk modification, the authors use the word “potentially.” In fact, reliable evidence of efficacy is lacking in plastic surgery patients. A prospective series of 1000 consecutive plastic surgery outpatients screened with Doppler ultrasound examinations before and after surgery revealed that hormone supplementation does not increase VTE risk [4]. This is an important finding for several reasons. One is that stopping birth control is not innocuous. This practice will inevitably lead to unplanned pregnancies, increasing VTE risk [5]. Cessation of hormone supplementation in menopausal women will exacerbate their symptoms. Fortunately, most plastic surgeons do not insist upon this unnecessary measure [1]. The only significant risk factor for VTE in plastic surgery outpatients that persists on regression analysis is patient age [4], which, of course, is not modifiable.

The authors comment that the baseline VTE risk level among the breast and body aesthetic population is unknown [1]. In fact, the VTE risk for outpatient aesthetic procedures has been determined in 1000 cosmetic surgery outpatients, including face, body, and breast procedures [4]. The overall VTE risk is 0.9%.

The authors also apply a Monte Carlo simulation to abdominoplasty outpatients [1]. Unfortunately, they do not have a similar series of 100 abdominoplasty patients with Caprini scores to determine their distribution in abdominoplasty patients. Instead, the authors applied the simulation to a very large study of outpatient abdominoplasty cases reported by Keyes et al. [6]. Among 240 abdominoplasties complicated by VTEs, 200 had sufficient information to calculate Caprini scores. Keyes et al. [6] concluded that Caprini scores were unhelpful because 89% of abdominoplasty patients who developed VTEs had Caprini scores ≤ 6.

The national web-based database used by Keyes et al. [6] did not include Caprini scores for abdominoplasty patients who did not develop VTEs. Pannucci et al. [1] decided to “backfit” their analysis. The authors applied the same 5.96-fold risk increase for patients with Caprini scores ≥ 7. To accommodate their backfit, the authors determined that 98% of the abdominoplasty patient base likely had Caprini scores ≤ 6 [1]. Such a very high percentage of patients with low Caprini scores is much different from the 81% prevalence of Caprini scores ≤ 6 (1519/1875 patients) [1] among nonanticoagulated VTE Prevention study plastic surgery inpatients.

In the absence of face-to-face baseline Caprini score data, such statistical retrofits are risky. It is not clear why abdominoplasty patients would have even lower Caprini scores (98% ≤ 6) than breast augmentation patients (96% ≤ 6), who tend to be younger, leaner, have fewer varicose veins, and undergo much shorter procedures. These differences would give many breast augmentation patients a 3- to 4-point advantage [7].

Although the authors used a Monte Carlo simulation, it is possible to make simple calculations to determine risk (Table 1). Such a calculation produces a 76% estimate for the percentage of breast augmentation patients who develop VTEs with Caprini scores ≤ 6. For abdominoplasty patients, a value of 98% is calculated for the proportion of all patients with Caprini scores ≤ 6. These values closely approximate the authors’ simulations (80% and 98%, respectively). When the calculation is done without a 6.0-fold risk multiplier, the percentage of abdominoplasty patients with Caprini scores ≤ 6 drops to 89%, which may be more realistic for this population. When the risk multiplier is not used for breast augmentation patients, the prevalence of Caprini scores ≤ 6 remains 96%, as expected for young, healthy, nonobese cosmetic surgery patients undergoing short procedures. Interestingly, the estimated VTE risk does not affect these percentages (Table 1), except for VTE risk estimates for patients with Caprini scores ≥ 7, which remain low for each patient group (< 1.2%).

**Table 1.** Venous thromboembolism (VTE) risk and Caprini scores in breast augmentation and abdominoplasty patients

Procedure*	Estimated VTE risk (%)	Number of patients	Risk multiplier	Patients with Caprini scores ≥ 7	Total patients with VTEs	Patients with VTEs with Caprini scores ≥ 7	VTE risk for patients with Caprini scores ≥ 7 (%)
Breast augmentation simulation [1]	0.01	1,000,000	6.0	40,000 (4%)	100	24 (24%)	0.06
Breast augmentation simulation [1], no risk multiplier	0.01	1,000,000	1.0	40,000 (4%)	100	4 (4%)	0.01
Abdominoplasty simulation [1], 0.01% VTE risk	0.01	1,000,000	6.0	20,000 (2%)	100	12 (12%)	0.06
Abdominoplasty simulation [1], 0.20% VTE risk	0.20	1,000,000	6.0	20,000 (2%)	2000	240 (12%)	1.2
Abdominoplasty, Keyes et al [6], 0.07% VTE risk	0.07	1,000,000	6.0	**18,000 (1.8%)**	700	77 (11%)	0.42
Abdominoplasty, Keyes et al [6], 0.01% VTE risk	0.01	1,000,000	6.0	**18,000 (1.8%)**	100	11 (11%)	0.06
Abdominoplasty, Keyes et al [6], no risk multiplier	0.07	1,000,000	1.0	**110,000 (11%)†**	700	77 (11%)	0.07

^*^Groups are subdivided by estimated VTE risk and whether or not a risk multiplier is applied for patients with Caprini scores ≥ 7. Note that the estimated VTE risk does not affect the percentage of patients with Caprini scores ≥ 7.†Without a risk multiplier, the calculated percentage of abdominoplasty patients with Caprini scores ≥ 7 is 11%.

Calculating a Caprini score requires assessment of 40 factors [7]. As a practical matter, compliance will always be an issue [5], and should be. Plastic surgeons’ time is valuable, and there are better uses for it than questioning patients about their history of any stillbirths, a sensitive question and one likely to stir traumatic memories. Lost pregnancies can hardly be considered a “modifiable risk factor or a risk factor potentially benefiting from further investigation” [1]. Do the authors recommend that patients have their varicose veins, a common and supposedly modifiable risk factor [1], treated before undergoing breast augmentation?

Pannucci [2] has previously claimed that “patients at high risk for VTE, specifically those with Caprini scores of 7–8 or > 8, have significant VTE risk reduction with provision of chemical prophylaxis.” The authors state that inpatient plastic surgery and inpatient surgery data suggest that plastic surgery patients with Caprini scores ≥ 7 benefit from anticoagulation, referencing two studies by the same lead author [3, 8].

The first referenced study is the 2011 VTE Prevention study [3]. In this study, the authors compared the risk of VTE in patients receiving enoxaparin with a historical control group of patients that did not receive anticoagulation. There was no significant difference in VTE risk comparing patients with Caprini scores of 7 or 8 (*P* = 0.230).

**Fig. 1.**
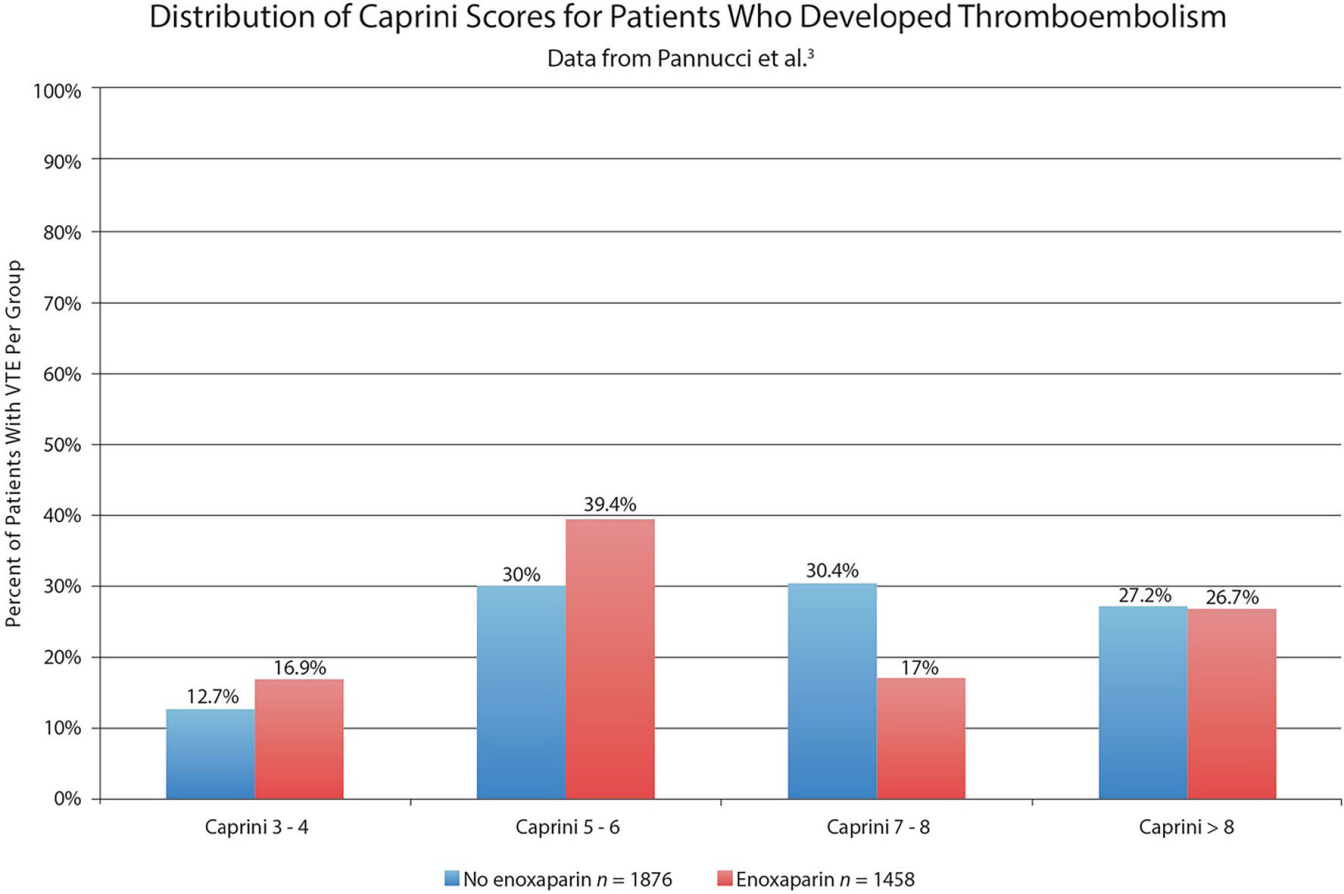
Histogram showing the distribution of affected patients by Caprini group. Unlike the authors’ graph [3], this is a true histogram; the percentages add up to 100%. The graph accounts for the 29% difference in sample sizes. A very similar amount of blue and red reflects the equal overall incidence of this complication. There is no evidence of a treatment benefit in patients with the highest Caprini scores (27% in both treated and untreated patients). Reproduced from Figure 5, Swanson [9]

Similarly, there was no significant difference in VTE risk for the cohort of patients with Caprini scores > 8 (*P* = 0.182) [3]. Scientifically, one cannot claim a treatment benefit (and certainly not a “significant” one) [2] when the difference is nonsignificant (Fig. 1) [9].

The second referenced study is a 2017 meta-analysis evaluating VTE risk in surgical and nonsurgical patients from a number of specialties [8]. The overall VTE risk for patients who did not receive chemoprophylaxis was 2.45%. Examination of the study data reveals a VTE risk of 4.37% among patients who received chemoprophylaxis (*P* < 0.0001). For patients with Caprini scores of 7 and 8, the VTE risks were 5.37% for patients receiving chemoprophylaxis versus 4.02% for untreated patients—no significant reduction for anticoagulated patients. Among patients with Caprini scores ≥ 5, the VTE risk was significantly greater (*P* < 0.001) for anticoagulated patients.

Moreover, a 2016 meta-analysis by the same lead author found no significant difference in VTE risk (*P* = 0.08) for plastic surgery inpatients when compared by Caprini scores, but a higher risk of bleeding (*P* = 0.02) in anticoagulated patients [10].

Perhaps these findings should not come as a surprise. Caprini scores were not developed scientifically. Caprini used intuition, experience, emotion, and logic in determining his scores, and received undisclosed payments from numerous anticoagulant manufacturers [5]. The Caprini scoring system was published in *Disease-A-Month* [7], a journal for primary care physicians. Only 24 references are cited [7] and no relative risk data are provided to support the point assignments [5]. When these scores are compared to relative risk values published in medical specialty journals, there is no significant correlation between relative risk values and Caprini scores [5].

The authors reference ultrasound examinations but question their relevance [1]. Doppler ultrasound examinations are the only means to reliably diagnose (and study) deep venous thromboses [4]. A skilled operator can perform a Doppler ultrasound screening examination in less than 20 minutes, probably not much longer than the time needed to evaluate a Caprini score. The value of Doppler ultrasound examinations is in identifying deep venous thromboses early, and initiating treatment early, to suppress their propagation into larger, proximal and much more dangerous thromboses [4]. Without such screening, it is possible for a thrombosis to develop undetected and only announce itself at the time of a fatal pulmonary embolism.

Today, national meetings of the American Society of Plastic Surgeons regularly include presentations on the use of ultrasound in plastic surgery offices. It is a natural extension to adopt this modality in patients for early VTE detection and treatment in affected individuals. Swanson has been performing screening ultrasound examinations on all plastic surgery outpatients since 2013. So far, not one breast augmentation patient among over 600 cases has demonstrated ultrasound findings of a deep venous thrombosis. No breast augmentation patient has shown clinical signs of a VTE. No patient has been instructed to interrupt hormonal supplementation or the use of oral contraceptives. All patients are treated under total intravenous anesthesia without muscle relaxation, which is known to reduce VTE risk [4, 9].

The best evidence suggests that the most effective way to reduce the risk of VTEs in breast augmentation patients is to use intravenous anesthesia and avoid muscle paralysis [4]. Smoking and body mass index are not independently related to VTE risk in plastic surgery outpatients [4].

The disclosure that Dr. Pannucci has performed expert witness work for deep venous thrombosis and pulmonary embolism in plastic surgery patients merits comment. Whether this testimony is for the plaintiff or defendant in a VTE case is highly relevant [9]. There is no stronger conflict of interest than having given sworn testimony claiming that individual risk stratification, chemoprophylaxis, and sequential compression devices represent the standard of care. Once a plaintiff’s expert has testified and influenced a high settlement or a judgment against a plastic surgeon (adding a second victim to the tragedy), it is impossible to later change course. Unfortunately, some plastic surgeons prescribe anticoagulation to all their abdominoplasty patients solely to reduce medicolegal liability, despite concerns regarding whether this practice is safe (i.e., bleeding risk), effective, and cost-effective. High level-of-evidence data are now available for plastic surgeons to defend their evidence-based practices [4, 5, 9].

In summary, it is time to recognize that Caprini scores are simply not helpful in plastic surgery. They waste the valuable time of health care personnel, who have enough documentation responsibilities to deal with already. Risk mitigation efforts (e.g., instructing patients to stop taking hormones or change their contraception method) have never been shown to be effective in reducing VTE risk in plastic surgery patients, and are not supported by the best available evidence [4]. Recommending that women withhold hormone treatment is arguably insensitive, in addition to being unnecessary. There is no need to deprive patients of the advantages of rectus diastasis repair or combined procedures [4]. Sequential compression devices have not been found to be helpful in plastic surgery outpatients [4]. Anesthetic considerations are relevant [4, 9]. Ultrasound surveillance offers a safe and effective method for VTE prevention in plastic surgery patients [4]. Technological advances in medicine are often met with resistance, but soon become routine.
